# Haplotype Affinities Resolve a Major Component of Goat (*Capra hircus*) MtDNA D-Loop Diversity and Reveal Specific Features of the Sardinian Stock

**DOI:** 10.1371/journal.pone.0030785

**Published:** 2012-02-17

**Authors:** Daniela Piras, Maria Grazia Doro, Giuseppina Casu, Paola Maria Melis, Simona Vaccargiu, Ignazio Piras, Debora Parracciani, Roberta Stradoni, Bruno Frongia, Graziano Lai, Salvatore Sale, Walter Cattari, Roberto Piras, Ombretta Querci, Piergiorgio Demuro, Sandro Cui, Franco Atzori, Marco Mancosu, Francesca Marchiori, Rossana Cammelli, Alessandra Spiga, Pier Paolo Loddo, Gianfranco Pili, Roberto Boi, Giuseppe Argiolas, Paolo Mereu, Giovanni Giuseppe Leoni, Salvatore Naitana, Mario Pirastu, Andrea Novelletto

**Affiliations:** 1 Institute of Population Genetics, National Research Council, Sassari, Italy; 2 Shardna Life Sciences, Cagliari, Italy; 3 Genetic Park of Ogliastra, Perdasdefogu, Italy; 4 ARAS, Regional Association of Sardinian Farmers, Cagliari, Italy; 5 Department of Physiological, Biochemical and Cell Sciences, University of Sassari, Sassari, Italy; 6 Faculty of Veterinary Sciences, University of Sassari, Sassari, Italy; 7 Department of Biology, University “Tor Vergata”, Rome, Italy; Ben-Gurion University of the Negev, Israel

## Abstract

Goat mtDNA haplogroup A is a poorly resolved lineage absorbing most of the overall diversity and is found in locations as distant as Eastern Asia and Southern Africa. Its phylogenetic dissection would cast light on an important portion of the spread of goat breeding. The aims of this work were 1) to provide an operational definition of meaningful mtDNA units within haplogroup A, 2) to investigate the mechanisms underlying the maintenance of diversity by considering the modes of selection operated by breeders and 3) to identify the peculiarities of Sardinian mtDNA types. We sequenced the mtDNA D-loop in a large sample of animals (1,591) which represents a non-trivial quota of the entire goat population of Sardinia. We found that Sardinia mirrors a large quota of mtDNA diversity of Western Eurasia in the number of variable sites, their mutational pattern and allele frequency. By using Bayesian analysis, a distance-based tree and a network analysis, we recognized demographically coherent groups of sequences identified by particular subsets of the variable positions. The results showed that this assignment system could be reproduced in other studies, capturing the greatest part of haplotype diversity.

We identified haplotype groups overrepresented in Sardinian goats as a result of founder effects. We found that breeders maintain diversity of matrilines most likely through equalization of the reproductive potential. Moreover, the relevant amount of inter-farm mtDNA diversity found does not increase proportionally with distance. Our results illustrate the effects of breeding practices on the composition of maternal gene pool and identify mtDNA types that may be considered in projects aimed at retrieving the maternal component of the oldest breeds of Sardinia.

## Introduction

The genetics of domestic goat, *Capra hircus*, has recently received considerable interest to understand various breeding aspects. As in other species, polymorphism of the mitochondrial (mtDNA) control region has been the tool of choice to initially describe the diversity of extant breeds and to reconstruct the places and timing of domestication [1], [2], [3], [4], [5], [6], [7], [8]. This portion of the genome fits all three main requirements to be an optimal marker, i.e. sufficient evolutionary conservation, variability and structuring across the range of the species, and rapid yet constant evolutionary rate [9]. Additional advantages consist of a high number of copies per cell thus making analyses possible even from tiny amounts of material, while its haploid state makes genotyping unambiguous also for the highly polymorphic hypervariable (HV) segment.

A first exploration of goat mtDNA diversity [2] revealed three divergent lineages which increased to six in a large series of samples collected worldwide [5]. These analyses took into account the phylogenetic relationships among mtDNA types as well as the parameters of the distributions of mismatches within and between lineages, as to reconstruct the past demography of the populations that contributed to the overall sample. This information was used to infer that the coexistence of diverging lineages probably reflected different domestication events [2], [9], associated with demographic expansions [5]. In fact, mtDNA types that remain confined into a captive population are likely to undergo a numeric expansion as a result of either the enhancement of breeding success associated with domestication *per se* (see Box 1 in ref. [9]) or following a parallel expansion of the breeders [10].

However, most sequences (91% of individuals worldwide and up to 100% in local samples - see Table 2 in ref. [5]) fell into a single major lineage, called haplogroup A. Carriers of sequences belonging to this lineage could be found in locations as distant as Eastern Asia and Southern Africa, including the whole of Europe, in line with the high portability/mobility of this species [10], [11]. Given that this is the most widespread and internally diverse haplogroup, its further phylogenetic dissection would cast light on an important portion of the spread of goat breeding, especially in the Western part of the Old World. Achievement of this goal has been hindered by the high mutation rate at some sites along the HV segment. This generates alleles equal in state along multiple lineages, producing distance measures with poor information content for a phylogenetic analysis.

One piece of evidence relevant to spot phylogenetically related groups of sequences is the relative abundance of each type. In fact, as the population grows, founder lineages increase in number faster than the accumulation of new mutations, and new mutants are numerically scanty [12]. This produces a so-called star phylogeny in which a central type is abundant and connected to many rarer types, each differing at one or few positions. The finding of such a pattern is thus evidence of phyletic relatedness through a reduced number of founders. This type of analysis inevitably entails a certain amount of redundancy in the sequencing effort and requires large sample sizes [13].

Recent views regard genetic diversity of livestock as an important resource (e.g. ref. [14] and references therein). Since genetic diversity is the basis for evolutionary potential, its immediate benefit in domesticated species is the preservation of a resistance response against parasites and diseases. While this holds for large and perhaps global scales, on a more local scale, interest is growing towards the identification, preservation and promotion of local peculiarities which may increase the cultural and commercial value of animals and their products [9].

The first evidences of an autochthonous population of Sardinian goats derived from the Filiestru Bonuighinu (Mara, Sassari) and Oliena caves [15], [16], [17] which can be associated with the spread of human Neolithic peoples and cultures [18], [19]. In the early Iron Age, the foundation of Phoenician colonies was accompanied by a further input of animals, but little is known on its impact on goat population [20]. During the Roman occupation, goat products (meat, milk and leather) were highly valued, promoting a careful breeding system that led to an appreciable increase in body size [21]. With the fall of the Roman Empire (476 AD), the goat population suffered a strong reduction, and during the Middle Ages (1000–1492 AD) the purported forests damage led to laws against goat breeding [21]. A precise description of morphological and distribution features within the island was provided by Cetti [22] at the end of 17^th^ century: the goats were located in North-Western (Nurra), South-Western (Iglesias and Teulada), but especially in Eastern Sardinia. In the 19^th^ century, changes in the domestic fauna occurrred through selection and breeding aimed at improving the local breeds. Following the rural exodus of the post-World War II years, a gradual but steady return to farming and goat breeding took place from the 1970's [23]. This was due to the discovery of the potential of this type of farming which can be carried out both extensively (in mountainous areas characterized by poor pastures) and intensively (with highly selected breeds). Goats facilitate human presence in the most marginal and disadvantaged areas by exploiting an otherwise unexploitable economic resource.

Currently, Sardinia hosts the largest goat population of any Italian region, with approximately 250,000 heads, about 23% of the total national stock [23]. The prevalent breed in Sardinia is called “Sarda” [24], and its main characteristics are the strong resistance to unfavourable environment and rough forages typical of the species [25], plus good longevity and fertility. Genetic features of the Sarda breed have been previously investigated with AFLPs and STRs markers [26], [27], [28], suggesting a high degree of differentiation with respect to some other Italian breeds. Within the island, the Sarda breed has been crossbred with goats of the Mediterranean area, especially with the Maltese breed [29].

In this work, we addressed the diversity of the mtDNA HVI segment in a large sample of domestic goats assembled with a fine-grained sampling scheme from all over Southern Sardinia. The startling amount of diversity represented in our large dataset prompted us to explore its features in an attempt to 1) provide an operational definition of meaningful phylogenetic mtDNA units which may serve for future research, 2) investigate the mechanisms underlying the maintenance of such a reservoir by testing hypotheses on stationarity of population size and on modes of selection operated by breeders and 3) identify those aspects which may deserve promotion to increase the value of the historically-rooted goat types by incorporating genetic information.

## Results

### Patterns of DNA diversity

**Figure 1:**
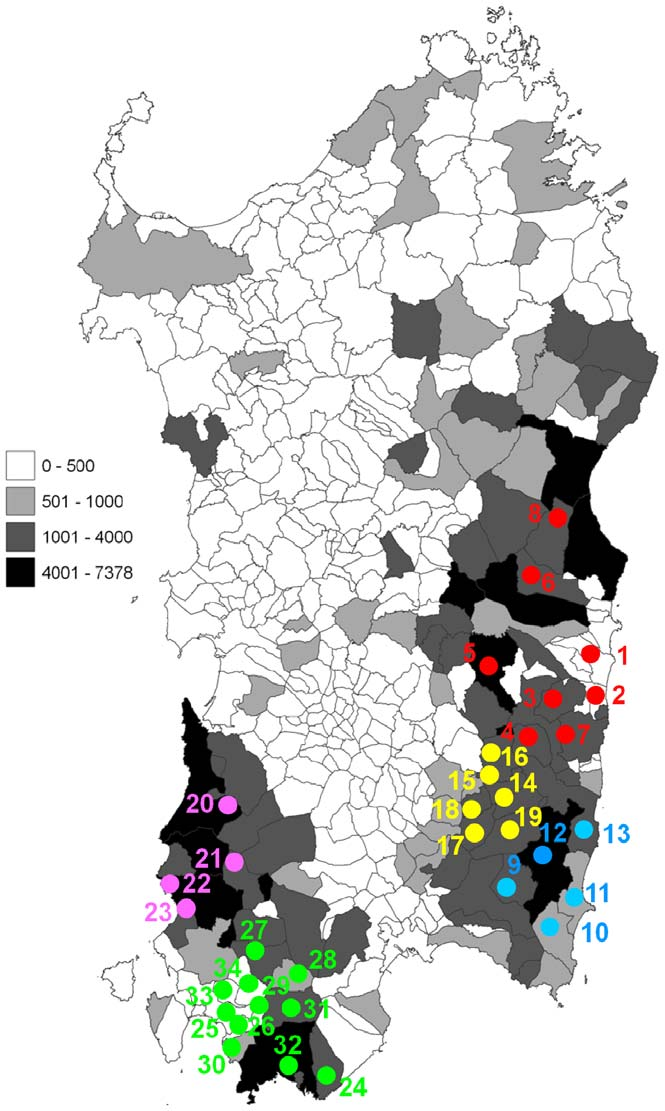
Map of Sardinia showing the 34 municipalities (dotted) where sampling was performed. In the background all municipalities are shaded according to the overall number of goats recorded [23], as reported in the left panel (map obtained at http://www.sar.sardegna.it/servizi/sit/datitematici.asp?wgs=1). Sampled municipalities in 5 Sardinian sub regions are shown with the following color codes: Ogliastra-red, Sarrabus-blue, Gerrei-yellow, Iglesiente-purple and Sulcis-green. Detailed names and n. of breeding stations sampled are reported in Table S1.

We sampled the Sardinian goat population from 5 Sardinian subregions comprising 1,591 specimens analyzed in the current study (Table 1, Fig. 1 and Table S1). The overall sequence length after alignment was 564 bp. In the set of 1,591 sequences obtained in this work (File S1), 166 variable positions were found. Allelic states at these positions are visually summarized in a file (File S2) also reporting the correspondence with the alignments obtained in previous works [1], [5].

**Table 1:** Composition of the sample of individuals yielding the set of mtDNA sequences used in all analyses, by provenance and breed.

	REGION						
BREED	GERREI	IGLESIENTE	OGLIASTRA	SARRABUS	SULCIS	Not recorded	Total
Not assigned	13	18	4	84	62		181
ALPINA			2		1		3
MALTESE	6	3	43	4	4		60
MALTESE+ALPINA			14				14
MALTESE+SAANEN			12		11		23
MALTESE+SPAGNOLA					1		1
MALTESE−SAANEN					1		1
SAANEN	2	2	13		9		26
SAANEN+ALPINA			11				11
SAANEN+SPAGNOLA					2		2
SARDA	105	51	470	33	257	8	924
SARDA+ALPINA			50		4		54
SARDA+MALTESE	56	17	87	16	69	6	251
SARDA+MALTESE+ALPINA					1		1
SARDA+SAANEN		5	12	1	2		20
SARDA+SAANEN+ALPINA					2		2
SARDA+SAANEN+SPAGNOLA					1		1
SARDA+SPAGNOLA	1				1		2
SPAGNOLA					14		14
Total	183	96	718	138	442	14	1591

Seven positions displayed 3 alternative nucleotides (268, 300, 347, 372, 376, 491 and 527). In all cases the third nucleotide was observed in a single instance. Of the remaining 159 positions, 50 displayed a singleton variant.

In 17 positions with two alleles, the substitution was a transversion. In all cases but one (pos. 458) these positions identified singleton variants.

Overall, 419 distinct mtDNA haplotypes were found in the 1,591 sequences (File S1). Their frequency spectrum is shown in Fig. S1. The haplotypes observed only once were 206. The most common haplotype was observed 79 times and was identical to those found in isolates SRI, SRH, SRA and M3 (Acc. FJ571580.1, FJ571579.1, FJ571572.1 and FJ571534.1) [6].

Haplotype diversity in the overall sample was 0.9899. Nucleotide diversity was 0.02199 per site, whereas the estimator Theta(S) was 20.63 over the entire sequence, or 0.042893 per site. Tajima's D was −1.366, statistically different from 0 (P = 0.04). This figure denotes that the frequency spectrum (Fig. S1) departed from neutrality for an excess of high and low frequency haplotypes, and a paucity of intermediate frequency haplotypes.

### Phylogenetic analyses

We first applied a Bayesian phylogenetic analysis to the set of 419 different haplotypes and an outgroup (AJ3178641) by using a model of nucleotide substitution that accomodates the unusual GC content of the mtDNA D-loop (HKY+gamma). The method has the advantage of estimating several parameters without conditioning on a single tree topology. In particular, we were interested in estimating the parameter of the gamma distribution describing the susbstitution rate heterogeneity among the 564 sites, as this can show how relevant homoplasy may be in confounding the phylogeny represented in our dataset [30]. Also, a low value may indicate relaxation of selective constraints and enhanced maintenance of mutants in the population [31]. Indeed we obtained a value of 0.168 (95% C.I. 0.158–0.180), lower than the previously reported values of 0.29 [2] and 0.28 [5].

The consensus of 2,251 trees sampled from the MCMC process (File S3), showed a single central node, from which 94 haplotypes and 38 clades (9 with credibility >95%) matching the majority rule departed.

While on the one hand this result showed that the phylogeny of our haplotype set could not be resolved to completion, on the other, the clear distinction of some subgroups and their replication in many trees encouraged us to search for an operational definition of meaningful mtDNA groupings. We thus explored additional methods to assay the robustness of haplotype clustering, in an attempt to produce a more manageable number of groups.

We constructed a Neighbour Joining (NJ) tree based on the Kimura 2-parameters distance, using a gamma correction with a value of 0.17 as obtained above (Fig. S2). Within the NJ tree we identified 12 major clades, which collectively grouped 409 of the 419 haplotypes (for a total of 1,567 out of 1,591 sequences). A reduced number of haplotypes (10 for a total of 24 sequences) escaped this classification. They are mainly isolated or small clusters of lineages, paraphyletic to the major clades.

For all haplotypes represented at least twice, we searched GenBank and the extensive dataset by Naderi et al. [5] to determine their haplogroup affiliation and the geographical region where identical sequences were found (detailed description as File S4). The main haplotype features and sequences falling within each clade are listed in Table 2.

**Table 2:** Properties of major mtDNA clades.

Clade	Total n. of sequences (% in haplogroup)	Total n. of different haplotypes	Defining sub-haplotype^a^	Entries of the 2×2 table^b^	r^2^	Mismatch observed mean	Raggedness index (p)	rho statistics±s.d.	Assigned in ref. [5] (% in haplogroup)	Assigned in ref. [1](% in haplogroup)
haplogroup C	12 (100)	7	Any of **155(G), 159(G), 279(G), 458(C)**	12, 0, 0, 1579	1.000	2.33	0.028 (n.s.)	2.42±1.22	30 (85.7)	0
A1	12 (2.9)	7	21(A)-115(T)-290(G)-**320(T)-380(T)**	12, 1, 0, 1578	0.923	2.83	0.042 (n.s.)	2.50±1.12	37 (1.7)	1 (1.7)
A2	195 (12.3)	36	**114(G)-**413(C)	188, 0, 7, 1396	0.959	2.03	0.036 (n.s.)	1.97±1.03	2 (0.1)	0
A3	83 (5.3)	14	104(C)-114(A)-**396(G)-413(C)**	83, 3, 0, 1505	0.963	2.09	0.035 (n.s.)	3.92±1.41	97 (4.4)	0
A4	336 (21.3)	77	**126(T)-348(C)-**415(T)	336, 0, 0, 1255	1.000	3.18	0.035 (<0.05)	2.17±0.54	2 (0.1)	0
A5	330 (20.9)	111	**42(G)-**104(C)-126(C)-**413(T)**	318, 29, 12, 1222	0.854	5.18	0.008 (n.s.)	4.74±1.35	631 (28.6)	7 (12.1)
A6	58 (3.7)	21	181(A)-**291(C)**	58, 0, 0, 1533	1.000	2.76	0.033 (n.s.)	2.43±0.96	101 (4.6)	0
A7	226 (14.3)	46	60(A)-100(T)-**104(T)-**291(T)-**343(A)**	224, 0, 2, 1356	0.990	2.53	0.044(c)	1.26±0.30	169 (7.7)	29 (50.0)
A8	104 (6.6)	17	60(A)-**104(T)-**181(A)-291(T)-343(G)	98, 33, 6, 1454	0.687	3.46	0.015 (n.s.)	3.51±1.31	1053 (47.7)	14 (24.1)
A9	29 (1.8)	12	181(A)-290(G)-291(T)-345(T)-389(A)-457(T)	28, 6, 1, 1556	0.792	5.68	0.051 (n.s.)	-	-	-
A10	46 (2.9)	11	**181(G)-**343(G)-389(A)	46, 0, 0, 1545	1.000	1.30	0.051 (n.s.)	0.72±0.32	6 (0.3)	2 (3.4)
A11	136 (8.6)	45	**60(G)-346(C)-**397(A)	136, 0, 0, 1455	1.000	3.34	0.037(c)	3.10±1.24	1 (<0.1)	0
Unclassified	24 (1.5)	10	n.a.	n.a.						
Total	1591	419							2099 (95.1)	53 (91.0)

a . Position in the alignment and allelic state (in bold = derived).

b . Entries of a 2×2 contingency table, in the order: With defining sub-haplotype AND In the clade, With defining sub-haplotype AND In other clades or unclassified, With other sub-haplotypes AND In the clade, With other sub-haplotypes AND In other clades or unclassified.

c . Not calculated, due to mismatch observed variance lower than mean [52].

The first clade (Fig. S2, bottom) grouped together 7 haplotypes (12 sequences). Sequences in this clade could be unambiguously assigned to haplogroup C and their closest neighbours were found in the subset of haplogroup C sequences sampled in France and Switzerland (99% similarity with Acc. nos. EF617786 and EF618486 [5]), whereas haplogroup C sequences found in Anatolian goats (e.g. HQ596552) had a higher number of nucleotide differences. This clade was the most deeply rooted in the tree and was highly supported by bootstrap (93%).

All other sequences were found affiliated with haplogroup A (branch support 99%) and the 11 clades were numbered A1 to A11, following a nomenclature with alternate letters and numbers (see e.g. ref. [32]). In general, the lengths of the basal branches were short and the nodes defining the 11 clades received a low bootstrap support, the highest being 59% for clade A11.

However, when this partition was compared with the one obtained with Bayesian inference, a highly overlapping pattern of haplotype grouping was observed (Table S2). In 35 out of 38 cases, sequences mapping to a given Bayesian clade mapped to a single NJ clade. The excepions were two haplotypes of NJ clade A8 which were grouped into Bay008 together with all haplotypes of NJ clade A10 (the latter forming a subclade with 92% credibility) and three haplotypes of NJ clade A9 which were grouped into Bay033 together with all haplotypes of NJ clade A11 (the latter forming a subclade with 93% credibility) . Finally, 5 NJ unclassified haplotypes were grouped into Bay020 with all haplotypes of NJ clade A1. It is noteworthy that the ungrouped 94 haplotypes in the Bayesian consensus tree were not evenly represented into NJ clades (penultimate line in Table S2). In particular, the highest proportion was lumped into NJ clade A5. Conversely, Bayesian analysis grouped exhaustively all haplotypes falling into NJ clades A1, A6, A7, A9, A10 and A11.

**Figure 2:**
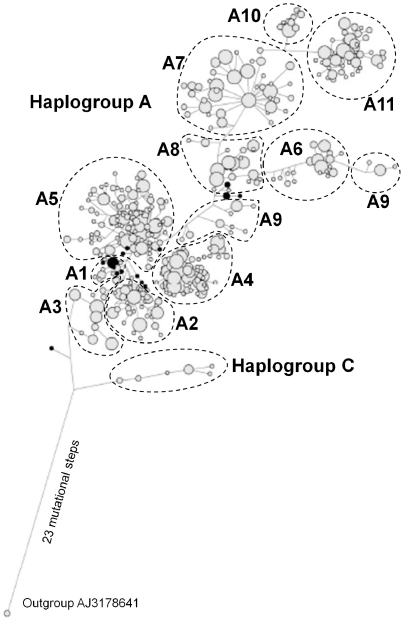
Median joining network of 419 haplotypes and an outgroup obtained as described in Materials and Methods. Branch length is not proportional to mutational steps. Node size is proportional to haplotype frequency. The correspondence between node clusters and clades in the NJ tree is reported. Unclassified sequences are blackened.

We used haplotype network analysis to further test the robustness of our haplotype groups and whether they could be taken as proxies of the overall phylogeny represented in our sequences. We then weighted each position according to the strength of haplotypic conservation by measuring linkage disequilibria between pairs of positions in the overall set of 1,591 sequences, and attributing to each position a proportionally increasing weight, as described in Materials and Methods. The network was characterized by distinct haplotype clusters (Fig. 2). Each of these largely overlapped one of the subclades of the NJ tree described above, with the exception of haplotypes classified into clade A9. Haplotypes of two of the A9 subclades (see above) were found directly linked to the torso of the network, while haplotypes of the third subclade formed a small separate cluster on the right side of the network.

Within the network, only the large cluster corresponding to clade A5 (Fig. 2) displayed a large number of reticulations. All other clusters showed a simple structure, with a central, highly frequent haplotype, and haplotypes with lower frequencies radiating from it, i.e. a star-like structure. Twenty-three mutations separated the outgroup from the first median node, linking haplotypes classified as haplogroup C. The second connecting cluster corresponded to clade A3 and this was in turn linked to haplotypes of clades A1 and A2. Clusters of haplotypes classified into clades A7–A10 formed the main torso of the network or departed from it. The most distal haplotypes were those assigned to clade A11.

### Hidden patterns of haplotypic conservation

The estimated gamma parameter and the reconstructed mutational events necessary to build the network, pointed out that a large number of highly mutable nucleotide positions add a great deal of noise in the phylogeny of haplogroup A, generating homoplasic allele states. In an attempt to reduce this complexity, we identified the particular arrangements of allele states, or sub-haplotypes that best identified each NJ clade or network cluster. The results are reported in cols. 4–6 of Table 2 which describe the sub-haplotype and its degree of association with the clade, as measured by the squared correlation coefficient in a 2×2 contingency table. Note that a strong association of this kind is also a good indication of haplotypic conservation because, by identifying groups of sequences with low Kimura's distances, positions that define affiliation to each group become markers of similarity at the array of all remaining positions.

As expected, haplogroup C was the most clearly defined group, with derived states at four positions, each of which uniquely identified this haplogroup among those here found. Among the 11 haplogroup A clades, four displayed a perfect (r^2^ = 1.0) correspondence with a particular sub-haplotype and five displayed an r^2^>0.85. A low r^2^ value was observed for clade A9, in line with its internal heterogeneity.

Two features emerged. First, all sub-haplotypes, except A9, included at least a derived allele which strongly contributed to define the clade. Second, some positions recurred in the listing, with ancestral and derived alleles. (e.g. pos. 60, 104, 181, 343), suggesting that these sites may mark the main phylogenetic relationships within haplogroup A. In particular, a single occurrence of an A>G mutation was invoked in the network at pos. 60, which then specifically identified clade A11, and was additionally in strong disequilibrium with 346(C) and 397(A).

In order to validate the above definitions, we used the same arrays of alleles to assign the sequences of two external datasets [1], [5]. This procedure was not applied for clade A9, in view of the lack of derived alleles. The results (Table 2, last two cols.) showed that this assignment system could be reproduced and captured the greatest part of haplotype diversity. In particular, in the two datasets, only 126/2,208 [5] and 5/58 [1] sequences could not be assigned, respectively, and 13/2,208 [5] and 0/58 [1] received an ambiguous (double) assignment.

In summary, the above analyses revealed that three clades are definitely overrepresented among Sardinian goats, i.e. A2, A4 and A11, for which a founder effect is the most likely explanation.

### Demography and population structuring

Figure S3 displays the distributions of pairwise mismatches for the entire set of sequences and each clade, while average values are in col. 7 of Table 2. When all sequences were considered, the distribution showed an average of 10.6, very similar to previous figures for global samples of haplogroup A [2], [5], which dominated our data, while the same average ranked at the top of the list of values for local samples [1]. Haplogroup C showed an unimodal distribution with a much lower mean than previously reported [2], [5]. This is in line with the origin of our haplogroup C sequences from the Western part only of the home range of haplogroup C.

The overall distribution showed a secondary mode for low values which did not disappear when only haplogroup A sequences were considered (mean = 10.2), thus indicating that some recent expansions affected one or more subsets of haplogroup A in our dataset. In all cases, small mismatch averages were observed, and the hypothesis that the observed distribution was drawn from a population which suffered a sudden expansion could be rejected only for clade A4 (Table 2, col. 8). We also calculated the rho statistics for all clusters except those corresponding to clade A9 (Table 2, col. 9). There was a strong correlation between the mismatch means and the rho statistics (Fig. S4), with clade A3 as the only outlier. In fact, in this cluster the most common haplotype (n = 40) is 4 mutational steps from the first haplotype encountered in the network path. This, and the ragged mismatch distribution, converged in supporting the idea that this cluster had a complex demographic history.

We used AMOVA to analyse the overall structuring within our series. We then partitioned the total diversity according to different criteria (Table 3). In this analysis inter-haplotypic variation is weighted according to the number of nucleotide positions differentiating them, but without directly addressing the phylogenetic relatedness between them, i.e. the informativeness of the positions in the phylogeny.

**Table 3:** Analysis of MOlecular VAriance after partitioning according to different criteria.

Partitioning criterion	n. of groups	n. of individuals	Fixation index	P
Geography	5 geographic regions	1,577 with known region	Fst = 0.0108	<1E-4
Breed	5 breeds	1,027 with unambiguous assignment	Fst = 0.0188	0.0109
Breeder	120 breeding stations	1,591	Fst = 0.1033	<1E-4
Breeder & Geography	119 breeding stations into 5 regions	1,577 with known region	Fsc = 0.0999; Fct = 0.0062	<1E-4 0.0068
Breeder in individual regions				
GERREI	14 stations	183	Fst = 0.0599	<1E-4
IGLESIENTE	8 stations	96	Fst = 0.1378	0.0004
OGLIASTRA	48 stations	718	Fst = 0.1250	<1E-4
SARRABUS	12 stations	138	Fst = 0.0534	<1E-4
SULCIS	37 stations	442	Fst = 0.0837	<1E-4

Geography alone explained only a minor quota of differentiation which was nevertheless significant in view of the overall sample size.

Partitioning according to breed (applied only to individuals with unambiguous assignment) also revealed a low degree of differentiation (Table S3).

Conversely, the differentiation across breeding stations was much stronger, with a 10 fold increase in the fixation index (Table 3, line 4). We explored more in detail the effect of geographical distance by considering individual regions, where breeding stations are often within walking distance from each other. We found large Fst values within all regions, with the highest values in the mountainous Iglesiente and Ogliastra, and only a minor increase in diversity across regions (Fct in Table 3, line 5). The idea of high connectivity between closely spaced breeding stations decreasing with increasing distance thus finds little support.

**Figure 3:**
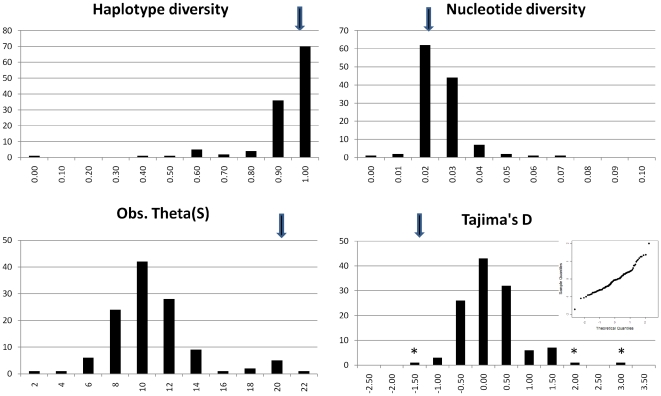
Histograms of four summary statistics for DNA diversity from 120 goat breeding stations. Black arrows point to the value obtained in the overall dataset. In the Tajima's D histogram, significant (nominal p<0.05) values are marked with an asterisk and a normal QQ plot is shown in the inset.

The above results fall between two possible extremes determined by the mode and strength of selection operated by breeders, at least on maternal lineages. At one extreme, the selection of one or few lineages, favoured by the reduced number of individuals at each breeding station, their relative isolation and the short generation time may produce the confinement of diverging lineages and high fixation indexes. At the other extreme, the maintenance of diversity is potentially able to equalize the mtDNA pools among stations, producing very low fixation indexes. In order to test which of the two modes could better explain our data, we analysed the distributions of summary statistics obtained in the 120 breeding stations (Fig. 3). In 70 out of 120 stations (58%) a diversity above 0.90 was retained, i.e. a value comparable to that found in the entire series. Also nucleotide diversity was largely maintained, with 106/120 stations (88%) producing a value between 0.01 and 0.03, as compared to 0.022 in the entire dataset. Conversely, the estimator Theta(S), based on the number of segregating sites, showed a marked reduction, dropping from 20, observed in the overall series, to a mode between 8 and 10 in the distribution of individual values. Contrary to Theta(S), this effect is attributable to the reduction of singletons or low frequency sites within each station, which affected only minimally both haplotype diversity and nucleotide diversity. As a result, the Tajima's D distribution underwent a shift toward small values (around 0, with no significant values after correcting for multiple tests), which testifies for an increase of intermediate frequency alleles within each station.

These results indicated that the breeding practice not only does not favour a single mtDNA type, but maintains and promotes diversity of mtDNA types (Table S3); no evidence of purification emerged for bodily traits either (Table S4). Out of the 110 stations where diagnostic breed features were recorded, 69 harboured two or more phenotypes. Whether the preservation of such phenotypic diversity is dictated by the adaptive value of particular anatomic, behavioural or productive features of individuals from different breeds or is coincidental, remains to be determined.

## Discussion

We explored the mtDNA D-loop variation in a large sample of animals grown by breeders in the entire Southern part of the island of Sardinia by examining a DNA region exactly overlapping previous studies. Our sample represents a non-trivial quota of the entire population of the island [23]. Despite the relative geographic isolation, we did find an amount of diversity that surprisingly summarizes a large quota of worldwide variation, as testified by summary statistics (see the listing in Table 1 of ref. [1]). Within our set we recognized demographically coherent groups of similar sequences that also recur in other studies and that may guide future sequencing efforts aimed at resolving the phylogeny of the whole haplogroup A. Our results also illustrate the effects of breeding practices on the composition of the maternal gene pool and identify mtDNA types that may be considered in projects aimed at retrieving the maternal component of the oldest breeds grown in Sardinia.

As far as phylogeny is concerned, Naderi et al. [5] introduced the term haplogroup to indicate six deep-rooted major lineages in mtDNA. We found representatives of haplogroup C, an old lineage [2] whose presence in Sardinia is to be attributed to its introduction from the Central-Southern European stocks. All the remaining haplotypes belonged to the almost ubiquitous and poorly resolved haplogroup A. In worldwide data, haplogroup C turned out to be less diverse than haplogroup A and was considered the result of an independent and more recent domestication event [2], [9]. In our data, haplogroup C displayed a reduced mean of the mismatch distribution, whereas haplogroup A produced a value overlapping the general one. We took this as evidence for multiple imports of haplogroup A haplotypes into the Sardinian stock. Its composition, therefore, replicates at least the Western Eurasian gene pool, thus making our analysis of a broader value.

The overall pattern of haplotype diversity resulted in a reproducible clustering of similar haplotypes, except a substantial polytomy of major clades within haplogroup A, which will need further work to be resolved. Bayesian and distance-based analyses supported each other in identifying the same groups of similar haplotypes, but an important piece of evidence was added by network analysis which, by using the entire set of 1,591 sequences, revealed the demographic consistency of most groups. This represented a step forward in reducing the overall complexity of haplogroup A [2], [5]. With the operational definition here given, based on allele arrangements at selected positions (Table 2), the 11 NJ clades approached the conditions of exhaustiveness and mutual exclusion in our dataset and the datasets of others [1], [5]. The observation of such a conserved Linkage Disequilibrium pattern in the deep ancestry represented in the three data sets suggests a common underlying phylogeny. The putative founders of these groups, identified as the modal haplotype(s) within each group, may serve to work out the real phylogenetic relationships between deeper branches within haplogroup A by exploiting variation in wider portions of the mitochondrial genome. Also they may complement the listing of reference sequences (Table 5 in ref. [5]), which currently lacks representatives of clades A2–A4, A6, A8 and A11.

As far as demography is concerned, most of the haplotype groupings displayed the landmarks of demographic growth. We refrained here in translating the mismatch averages into dates for the beginning of the expansions, in view of the large uncertainty on average and site-specific mutation rates. However, given the widely discrepant frequencies of each group in and out Sardinia, the overall haplotype distribution in the island is best explained by two overlapping processes: i) expansions driven by local pastoralism (for clades A2 and A11) and ii) massive imports which reflect clade expansions occurred elsewhere [33].

Luikart et al. [2] pointed out that, historically, goats have been a portable asset which represented a smaller currency unit as compared to cattle, two factors that favored its spread in remote areas including transfer by maritime transport [1], [19]. In Sardinia its successful settlement was further promoted by the particular climate and environmental conditions. Here, goats may have also been useful in clearing the almost ubiquitous dense shrub maquis [11]. In line with this, we found a very low level of geographical structuring. In addition, previous surveys reported a quota of variation between breeds of about 10% for mtDNA [2], [5] and of 6% for autosomal genes [34], much larger than that found by us. Both findings can be easily accounted for by the narrower spectrum of breeds represented in Sardinia and the higher historical connectivity between breeding stations within the area surveyed in this study. In particular, this latter factor has most likely favoured the exchange of animals, reducing the anchoring of specific breeds to particular places (see Table S4). In addition, the transfer of sires reshuffles phenotypic traits, mostly encoded by the nuclear genome with respect to mtDNA.

Finally, we found that breeders maintain diversity rather than selecting matrilines. This was initially suggested by the low value of the gamma distribution parameter, which indicated that our geographically confined sample not only allows for the full spectrum of mutational heterogeneity among sites, but probably also for the conservation of variability generated at some hypermutable sites. The most likely mechanism by which this conservation is achieved is the equalization of the number of progeny across the reproducing females. In fact, the reduction of variance of progeny size is potentially able to increase the effective population size even above the census size (e.g. see Equation 10.4 in ref. [35] Ne = (4N−2)/(Vk+2), where Vk is the variance in progeny size).

The observed level of residual inter-station diversity is likely the result of a high initial diversity of founders for each herd, increased by new mutant types, but diluted by the ongoing exchange of animals between herds. Although we cannot say whether these opposing forces are at equilibrium, we excluded that animal trade operates in a distance-dependent manner, at least within the range of distances achievable in Sardinia (tenths or few hundreds of km). This contradicts findings from other regions where differences in the ethnic affiliation of breeders drive the phenomenon [36].

On the one hand, the maintenance of high within-station mtDNA diversity adds resilience to the overall Sardinian mtDNA gene pool (e.g. in case of an epidemic); on the other, however, the presence of representatives of multiple divergent mtDNA lineages within many stations may indicate a strong dilution of the typical Sardinian types. Thus, also for Sardinia the question [9] “what kind of diversity do we wish to conserve, and what demographic and genetic processes do we hope to facilitate in the future?” is appropriate. We identified at least two candidate clades which displayed a Sardinian-specific radiation. Whether or not their presence is a result of one or more founder effect(s), may be tested by examining both translocated stocks of so-called “pure Sarda” breed and by analyzing DNA from ancient remains [37]. Their diversity and frequency should be given consideration in conservation programmes (for a discussion see refs. [38], [39]). Appropriate incentive measures can be implemented to safeguard these mtDNA types, together with valuable characteristics matching the Sardinian environmental conditions and historical dairy techniques.

## Materials and Methods

### Observational studies and sampling

All activities carried out by the Regional Association of Sardinian Farmers (ARAS) officers, part of which related to this study, followed ethical guidelines for care and use of agricultural animals for research (EC Directive 86/609/EEC). For this study specific approval by a review board was not necessary, as none of the procedures used here met the criteria to define them as experiments according to Article 2 of the cited Directive. Details on care/welfare of animals are available in the File S5. Samples of whole blood were obtained from 1,694 individuals collected in 120 different breeding stations scattered across 34 municipalities of Southern Sardinia (Fig. 1 and Table S1). This sampling covered the 5 historical Sardinian subregions with the largest goat population. In particular, Ogliastra has about 15% of the Sardinian goat population. Blood aliquots used for DNA studies belonged to samples routinely collected during animal health surveys carried out by ARAS, and their collection did not cause any additional burden to the animals. The number of goats in each farm were about 100–150 and for each farm 15 blood samples on average were collected (about 10%). Each group of sampled individuals was representative of morphological features of the herd in the farm. Five breeds (Alpina, Maltese, Saanen, Sarda, Spagnola; for ref. see http://www.agraria.org/ovini.htm) could be identified among the sampled individuals. Additional individuals carried mixed traits (Table 1) leading to ambiguous classification while other individuals could not be definitely assigned. A database including individual measurements, life history and genetic data is being assembled and will soon be publicly available. Verbal informed consent was initially obtained from all farmers to perform observational and genetics studies, and their willingness to participate is testified by information provided to ARAS officers on a continuous basis during the following years.

### Experimental procedures

Total DNA was extracted from whole blood using the ActivePUre DNA purification system (5PRIME GmbH), following the manufacturer's instructions. A 481 bp fragment of the mtDNA D-loop (HVS-I) was amplified with primers described in ref. [5].

Amplification was in a 15 µl reaction volume containing 1 ng of DNA, 0.5 pmoles of each primer, 200 µM of each dNTPs, 1.5 ul 10× PCR buffer with 15 mM MgCl2, 0.5 U of Eurotaq (CELBIO). Amplification was carried out in a GeneAmp PCR system 9700 (Applied Biosystem) with the following thermal profile: initial denaturing at 96°C for 5′, 35 cycles at 95°C for 30″, 56°C for 30″, 72°C for 1′ and a final extension at 72°C for 10′. Five ul of PCR product were treated with 2 µl of exosap-it at 37° for 15′ and 80°C for 15′. Sequence reactions were performed with the ABI PRISM BigDye Terminator kit (v1.1, Applied Biosystem) using 1.7 pmoles of primer F at 96°C 10″, 55°C 5″ and 60°C 2′ for 25 cycles and products analyzed in an ABI PRISM 3130×L Genetic Analyzer. Electropherograms were visually inspected. The complete dataset consisted of 1,694 sequences.

### Pre-treatment of sequencing data

Inspection of the raw data identified 103 sequences with one or more ambiguous positions that were not further considered. The remaining 1,591 sequences were aligned with Clustal W with standard parameters as implemented at http://mobyle.pasteur.fr. Sequence AJ3178641 from *C. aegagrus* was included as outgroup [2]. The sequence EF6182311, carrying a 76 bp insertion, was also included to aid in the alignment. The overall alignment covered 564 bp. The first position was homologous to pos. 15,709 of the complete mitochondrial genome sequence GU_295658 [40] and to pos. 1 in ref. [5]. However, the numbering of the remaining positions was not identical, due to the different occurrence of small insertions and deletions in the two datasets (see File S2). The large insertion was found to occur at homologous positions.

Overall, 186 positions with variable nucleotides were found after alignment. Two insertions, occurring in sequences JN085944 and JN085635 at position 20 and in JN085796 at positions 430–433, respectively, were not considered. In sequences JN085934 and JN085829 an additional C was observed at the 5′end of the large inserted segment, bringing its total length to 77 bp. This position was also not considered.

In the text we used the term haplotype to indicate different arrangements of allele states (nucleotides) at all or a subset of the 186 variable positions.

All non-redundant sequences were submitted to GenBank (Acc. nos. JN085528 - JN085946).

### Data analysis

Parameters of gene diversity were obtained with the program DNAsp [41].

These included: haplotype diversity or the probability of picking two haplotypes different in state; nucleotide diversity, i.e. an estimator of the population parameter Theta ( = 4 Neu where Ne is the effective population size and u the mutation rate) based on the number of segregating sites and their allele frequency; the estimator Theta(S), based solely on the number of segregating sites; Tajima's D, a summary statistics for the entire frequency spectrum of variable sites which takes into account the difference between the two above estimators.

Bayesian phylogenetic analysis was carried out with MrBayes 3.1.2 [42], [43] as implemented at CIPRES (http://www.phylo.org) [44], considering each variant sequence only once (see below) and AJ3178641 as outgroup. Two runs were used, each consisting of 3,000,000 generations. Markov chain sampling was every 1,000 generations. The HKY [45] substitution model was used (Nst = 2), to account for different transition/transversion ratios as well as for base frequency inequalities which are quite strong in mtDNA, in general, and in the D-loop, in particular. In order to account for substitution rate heterogeneity among sites, the gamma distribution model was used. Overall the HKY+gamma model was chosen as a reliable compromise between simplistic assumptions and overparametrized models (for a discussion see the mrBayes program manual). Default values and prior distributions were used for all parameters, with the exception of the number of chains (8 used) and the prior distribution for the gamma parameter (Shapepr), which was restricted to UNIFORM (0.01, 1.00). At the end of the run acceptance rates for the moves in the “cold” chain were between 7.5 and 63% and the average standard deviation of split frequencies was 0.08. The two runs reached stationarity after 750 of 3,001 sampled steps (as defined in the program manual), and this value was then chosen as “burn-in”.

Distance-based trees were obtained and drawn with the program MEGA [46] on the same set of sequences.The Kimura's 2-parameters distance measure was used, which also accounts for different transition/transversion ratios. Bootstrap analysis was performed with 1,000 replicates.

In order to further confirm haplotype grouping to get direct information of which allele state contributed to aggregating sequences, as well as to visualize the radiation of haplotypes, we performed a network analysis with the program NETWORK [47]. The median joining algorithm was applied to the 419 groups of identical sequences, by considering the absolute number of sequences falling in each group. The sequence AJ3178641 was considered as an outgroup and used to infer ancestral and derived states in the binarization process. Three-state positions were excluded. The 77 bp insertion was encoded as a single position with mutational weight 99. Singleton positions were given a weight of 1. We reasoned that in the case of a non-recombining molecule, such as mtDNA, the decay of perfect association between variant alleles at different sites can be attributed solely to the appearance of alleles equal in state by recurrent mutation along different lineages. Thus the strength of association, or Linkage Disequilibrium (LD), between pairs of sites can be taken as a proxy for their stability in the evolutionary timescale [48]. In order to take advantage of this information, each variable position, except those mentioned above, was given a weight proportional to the degree of LD with other positions according to the following scaling: 5, 10, 20 and 50 for 0–49, 50–59, 60–79 and > = 80 significant (P = 0.05) pairwise LD values. Under these conditions the weighting value summarizes information from multiple sites, as recommended [49]. Also the method is insensitive to different properties of LD estimators, as it uses P values. The resulting network was manually rearranged by increasing the distance between nodes and node clusters.

We also calculated the rho statistics for different clusters . This measure corresponds to the average number of mutations across haplotypes sharing a given ancestor and is linearly related to coalescence time [50], [51] in star-like phylogenies. For each cluster in the network we assumed the first (in the direction from the outgroup to the opposite end) non-empty node lying on the torso of the network as the ancestral haplotype.

Apportionment of DNA diversity according to different criteria and the associated diversity indexes were obtained with the program Arlequin v. 3.0 [52]. This program was also used to group identical sequences, to test Tajima's D with 10,000 simulations and to obtain the matrix of LD between polymorphic nucleotide positions (see above). Arlequin was also used to obtain mismatch distributions, to calculate the raggedness index [12], [53] and to test (5,000 bootstrap replicates) whether an observed mismatch distribution was drawn from an expanded population (small raggedness index) or a stationary one (large raggedness index). In these analyses the 77 bp insertion was encoded as a single variable position.

Identity search was performed by BLAST (http://blast.ncbi.nlm.nih.gov/Blast.cgi), by limiting the search to *Capra hircus* (taxid:9925), and recording the % identity in the region overlapping our sequence used as query. In order to search for the smilarities between our sequences and those of ref. [5] Blast2seq was used by loading the entire dataset of these authors.

## Supporting Information

Figure S1 **Haplotype frequency spectrum among 1,591 mtDNA sequences.**
(TIF)

Figure S2 **NJ tree of 419 haplotypes and an outgroup obtained with the Kimura 2-parameter distance and a gamma parameter = 0.17.** The nomenclature of the major clades is in bold, with unclassified sequences indicated with their Id. Bootstrap values are in regular font.
(TIF)

Figure S3 **Mismatch distributions of sequences of haplogroups A and C and within each of the major clades of the NJ tree. Percent observations are on the Y axis.** Note the different scales. In parentheses the number of sequences contributing to each distribution.
(TIF)

Figure S4 **Scatterplot of mismatch mean vs. rho statistics values of** **Table 2** **.**
(TIF)

Table S1 **Geographic distribution of sampling locations.**
(DOC)

Table S2 **Comparison of sequence assignment in the Bayesian consensus and the NJ trees.**
(DOC)

Table S3 **Representation of sequences of different NJ major clades according to breed (only unambiguous breed assignment) and region.**
(DOC)

Table S4 **Table of goat breed by farm.**
(XLS)

File S1 **Aligned sequences corresponding to each of the 419 distinct haplotypes.** Each line reports the GenBank Acc. N., Sequence Id., the absolute haplotype frequency and its clade assignment in the NJ tree.
(XLS)

File S2 **Summary of states at the 166 variable positions in 1,591 sequences.** The top rows report the position numbering the alignment of this and other works. Yellow represents identity with the outgroup and blue an alternative allele state.
(XLS)

File S3 **Plain text representation of the consensus of 2,251 trees produced by mrBayes under the conditions reported in** **Materials and Methods**. Sequences are identified by their Id. and position in the original input file (in parentheses).
(TXT)

File S4 **Summary of GenBank searches for identity/similarity to the most frequent mtDNA haplotypes found in this work.**
(DOC)

File S5 **Details of animal husbandry.**
(DOC)
