# Supplementary figures and images for: Haplotype Affinities Resolve a Major Component of Goat (Capra hircus) MtDNA D-Loop Diversity and Reveal Specific Features of the Sardinian Stock
Source: PLoS One. 2012 Feb 17;7(2):e30785. doi: 10.1371/journal.pone.0030785 (PMC3281868; doi:10.1371/journal.pone.0030785)

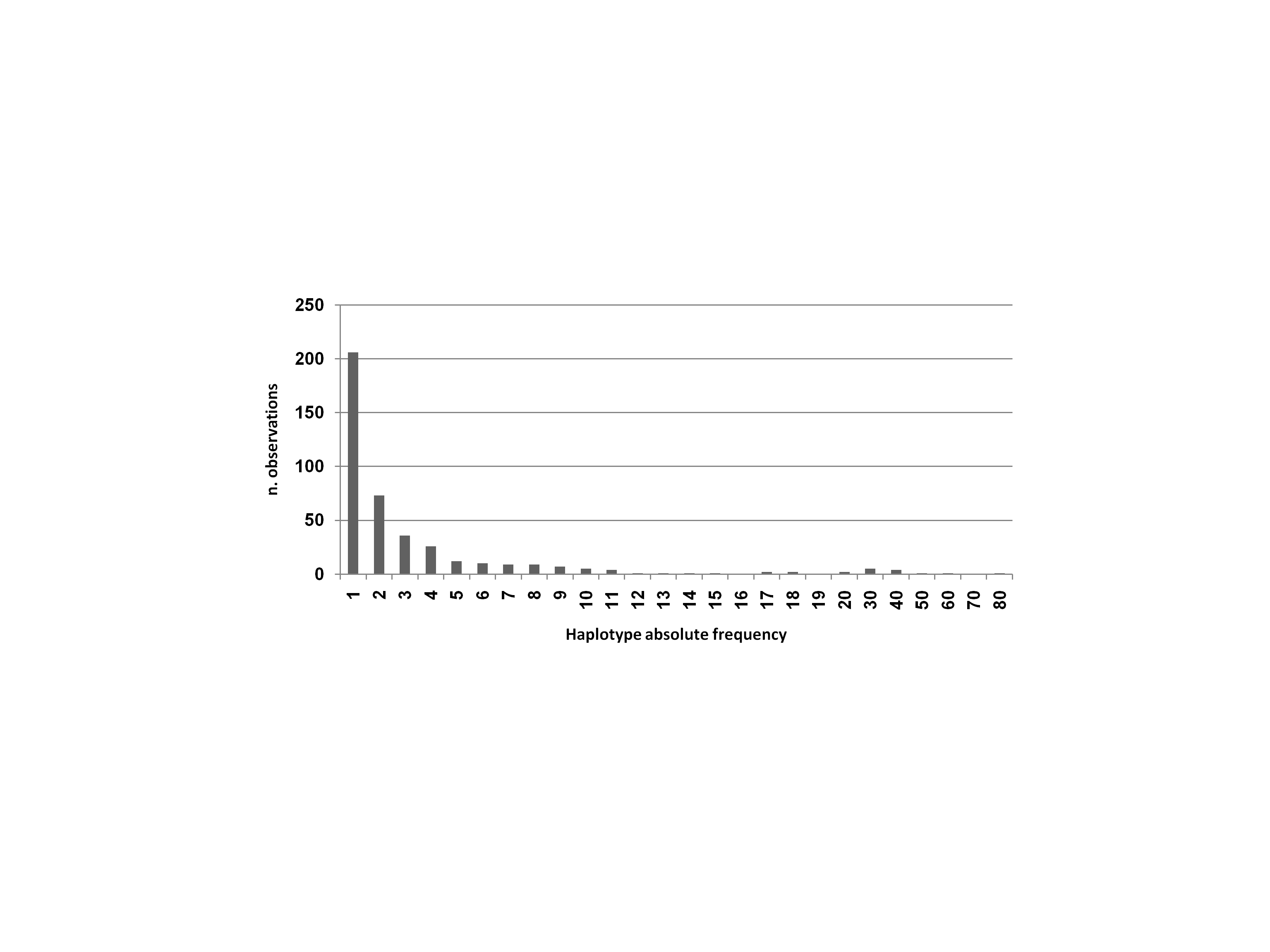

Supplement: Figure S1 — Haplotype frequency spectrum among 1,591 mtDNA sequences. (TIF) [file pone.0030785.s001.tif]

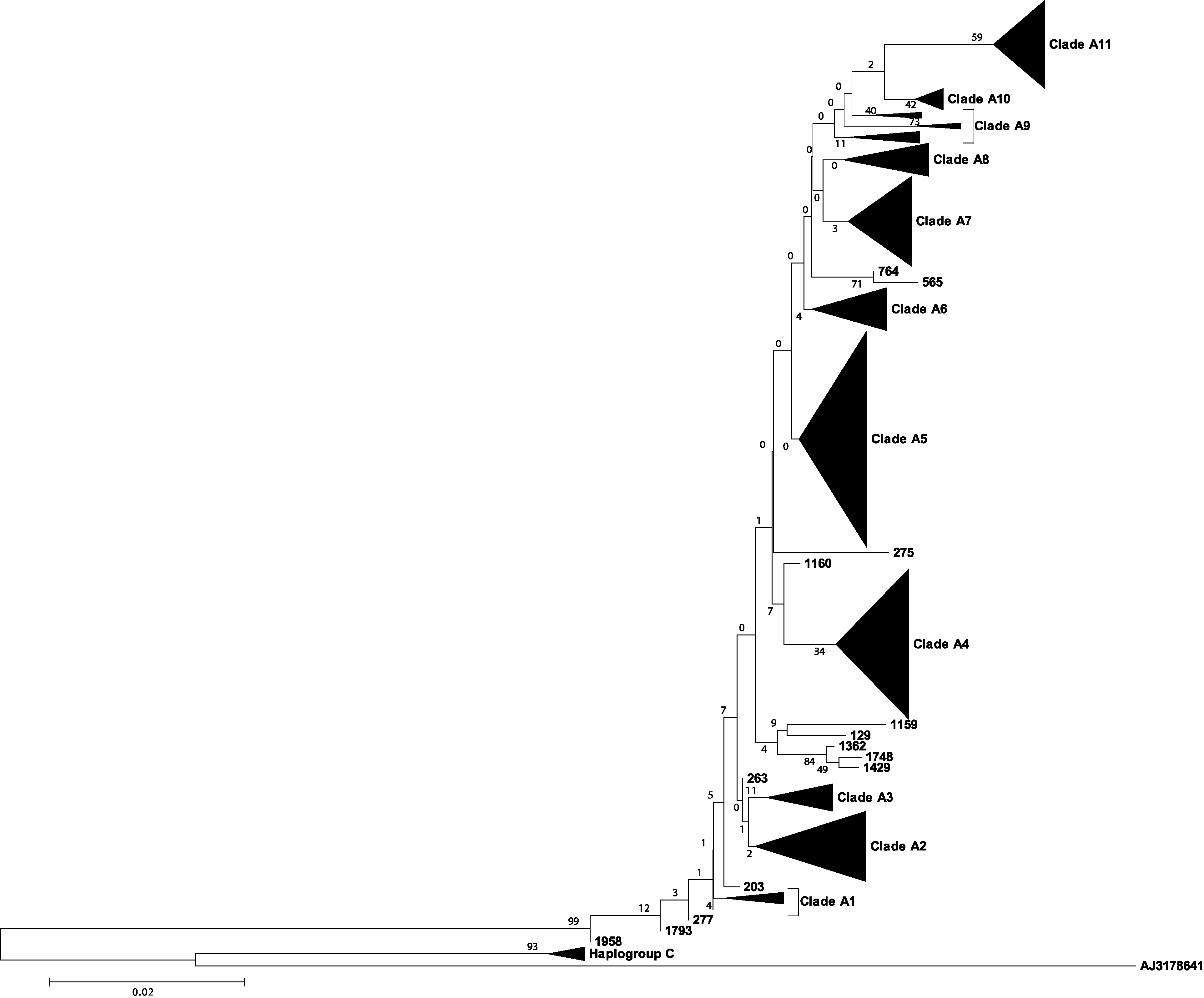

Supplement: Figure S2 — NJ tree of 419 haplotypes and an outgroup obtained with the Kimura 2-parameter distance and a gamma parameter = 0.17. The nomenclature of the major clades is in bold, with unclassified sequences indicated with their Id. Bootstrap values are in regular font. (TIF) [file pone.0030785.s002.tif]

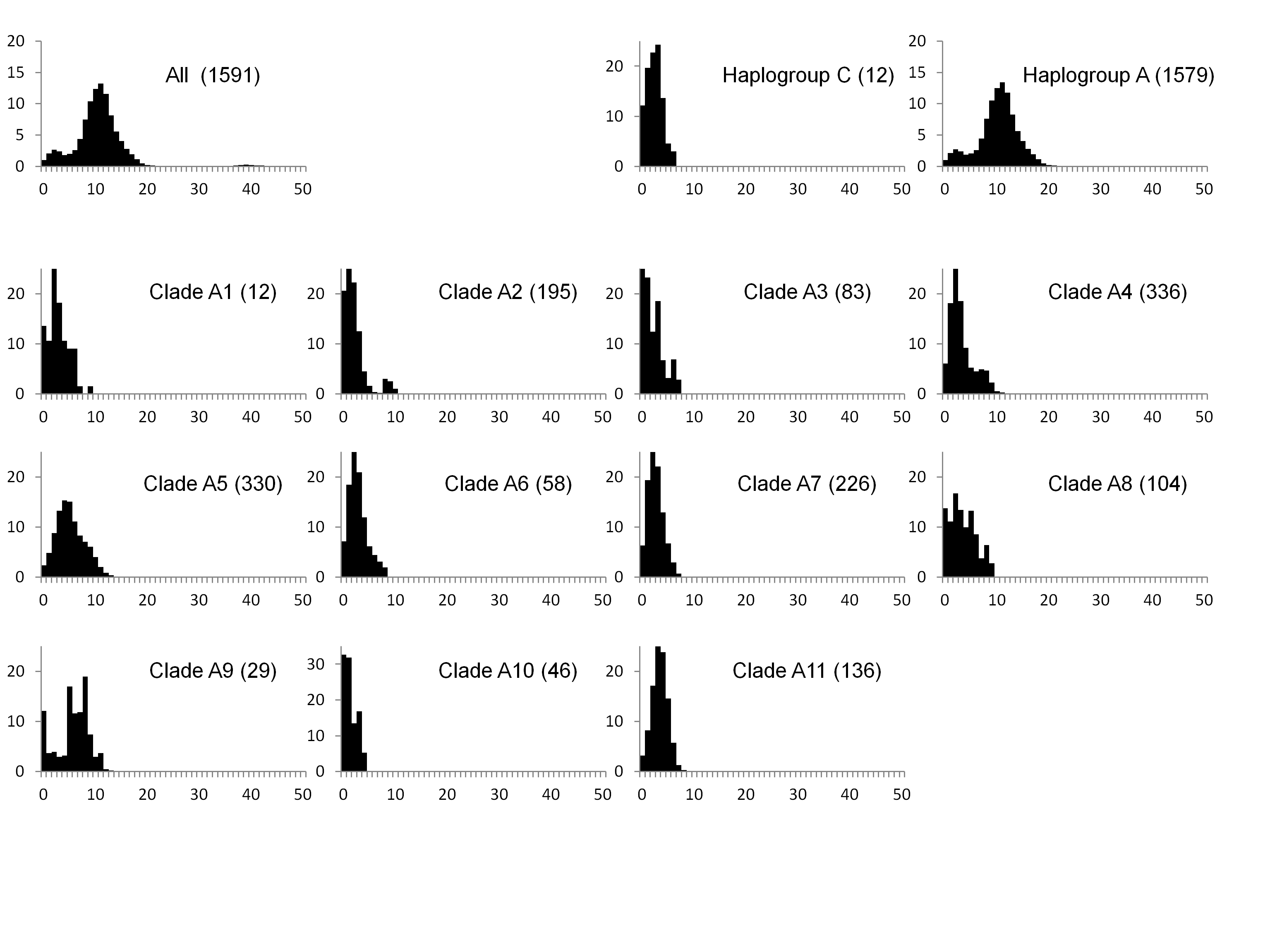

Supplement: Figure S3 — Mismatch distributions of sequences of haplogroups A and C and within each of the major clades of the NJ tree. Percent observations are on the Y axis. Note the different scales. In parentheses the number of sequences contributing to each distribution. (TIF) [file pone.0030785.s003.tif]

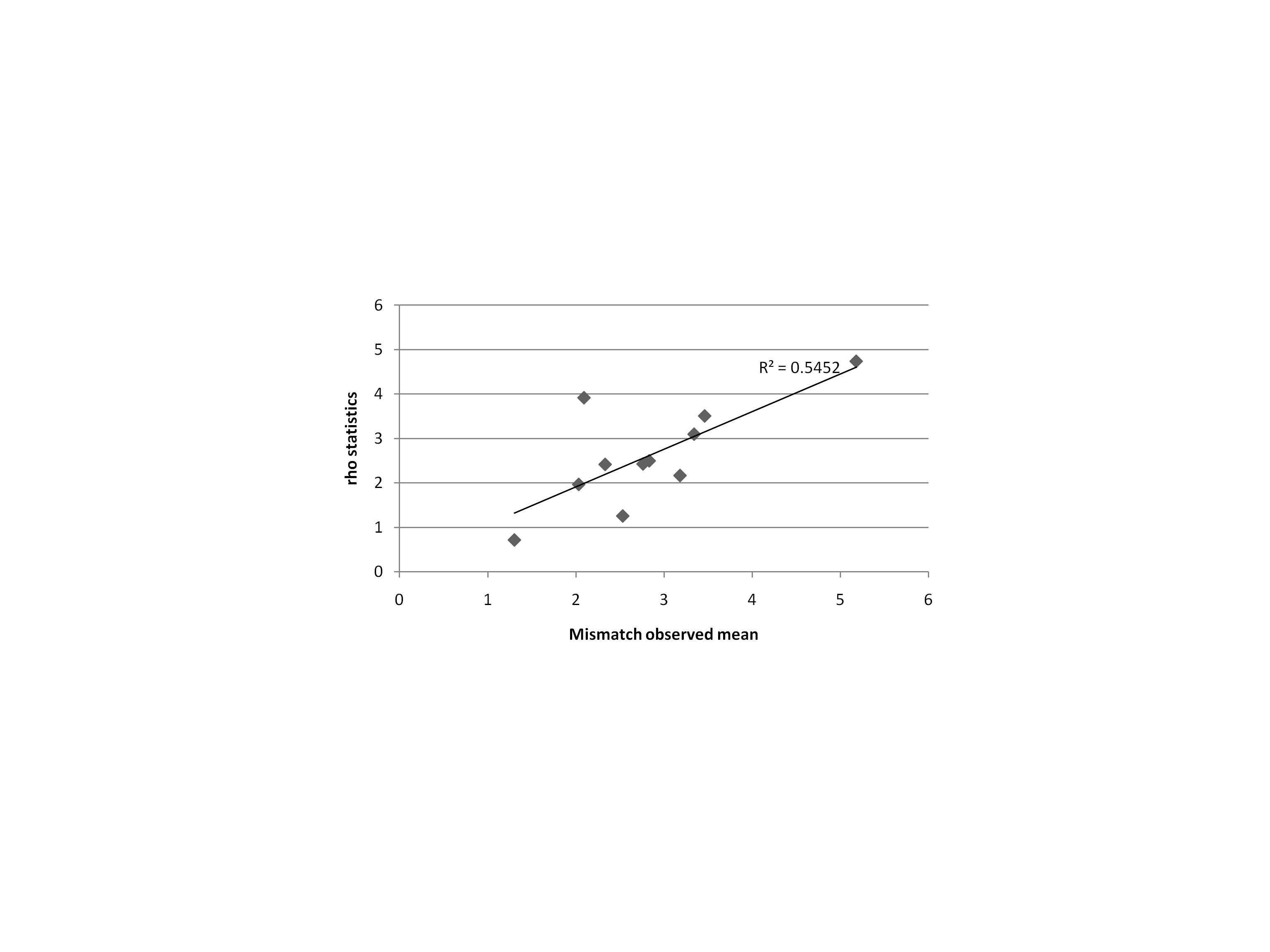

Supplement: Figure S4 — Scatterplot of mismatch mean vs. rho statistics values of Table 2 . (TIF) [file pone.0030785.s004.tif]
