# Supplementary material for: Haplotype Affinities Resolve a Major Component of Goat (Capra hircus) MtDNA D-Loop Diversity and Reveal Specific Features of the Sardinian Stock
Source: PLoS One. 2012 Feb 17;7(2):e30785. doi: 10.1371/journal.pone.0030785 (PMC3281868; doi:10.1371/journal.pone.0030785)
Supplement: Table S1 — Geographic distribution of sampling locations. (DOC) [file pone.0030785.s005.doc]

Supplementary Table 1. Geographic distribution of sampling locations

| Geographic subregion | n. in Fig. 1 | Municipality | n. of breeding stations |
| --- | --- | --- | --- |
| Ogliastra | 1 | Barisardo | 4 |
|  | 2 | Cardedu | 2 |
|  | 3 | Jerzu | 1 |
|  | 4 | Perdasdefogu | 8 |
|  | 5 | Seui | 5 |
|  | 6 | Talana | 16 |
|  | 7 | Tertenia | 1 |
|  | 8 | Urzulei | 11 |
| Sarrabus | 9 | Burcei | 1 |
|  | 10 | Castiadas | 4 |
|  | 11 | Muravera | 3 |
|  | 12 | S.Vito | 2 |
|  | 13 | Villaputzu | 2 |
| Gerrei | 14 | Armungia | 1 |
|  | 15 | Ballao | 1 |
|  | 16 | Escalaplano | 4 |
|  | 17 | S. Nicolò Gerrei | 1 |
|  | 18 | Silius | 2 |
|  | 19 | Villassalto | 5 |
| Iglesiente | 20 | Arbus | 2 |
|  | 21 | Domusnovas | 1 |
|  | 22 | Iglesias | 4 |
|  | 23 | S.Giorgio | 1 |
| Sulcis | 24 | Domus de Maria | 2 |
|  | 25 | Giba | 2 |
|  | 26 | Masainas | 2 |
|  | 27 | Narcao | 1 |
|  | 28 | Nuxis | 4 |
|  | 29 | Piscinas | 2 |
|  | 30 | S.AnnaArresi | 1 |
|  | 31 | Santadi | 11 |
|  | 32 | Teulada | 10 |
|  | 33 | Tratalias | 1 |
|  | 34 | Villaperuccio | 2 |
| TOTAL |  |  | 120 |
