# Supplementary material for: Haplotype Affinities Resolve a Major Component of Goat (Capra hircus) MtDNA D-Loop Diversity and Reveal Specific Features of the Sardinian Stock
Source: PLoS One. 2012 Feb 17;7(2):e30785. doi: 10.1371/journal.pone.0030785 (PMC3281868; doi:10.1371/journal.pone.0030785)
Supplement: Table S2 — Comparison of sequence assignment in the Bayesian consensus and the NJ trees. (DOC) [file pone.0030785.s006.doc]

Supplementary Table 2. Comparison of sequence assignment in the Bayesian consensus and the NJ trees.

|  | NJ clade^1^ | | | | | | | | | | | | |  |
| --- | --- | --- | --- | --- | --- | --- | --- | --- | --- | --- | --- | --- | --- | --- |
| Clade in Bayesian consensus  tree^2^ | Hg C | A1 | A2 | A3 | A4 | A5 | A6 | A7 | A8 | A9 | A10 | A11 | Uncl. | Total |
| Bay001 |  |  |  |  |  | 3 |  |  |  |  |  |  |  | 3 |
| Bay002 |  |  |  |  |  |  |  |  |  |  |  |  | 2 | 2 |
| Bay003 |  |  |  |  |  |  |  |  | 3 |  |  |  |  | 3 |
| Bay004 |  |  |  |  |  | 3 |  |  |  |  |  |  |  | 3 |
| Bay005 |  |  |  |  | 2 |  |  |  |  |  |  |  |  | 2 |
| Bay006 |  |  |  |  |  |  |  | 5 |  |  |  |  |  | 5 |
| Bay007 |  |  |  |  |  |  | 21 |  |  |  |  |  |  | 21 |
| Bay008 |  |  |  |  |  |  |  |  | 2 |  | 11 |  |  | 13 |
| Bay009 |  |  |  |  | 15 |  |  |  |  |  |  |  |  | 15 |
| Bay010 |  |  |  |  | 3 |  |  |  |  |  |  |  |  | 3 |
| Bay011 |  |  |  | 4 |  |  |  |  |  |  |  |  |  | 4 |
| Bay012 |  |  |  |  |  |  |  |  |  |  |  |  | 3 | 3 |
| Bay013 |  |  |  |  |  | 4 |  |  |  |  |  |  |  | 4 |
| Bay014 |  |  |  |  |  |  |  |  | 2 |  |  |  |  | 2 |
| Bay015 |  |  |  |  |  | 4 |  |  |  |  |  |  |  | 4 |
| Bay016 |  |  | 35 |  |  |  |  |  |  |  |  |  |  | 35 |
| Bay017 |  |  |  |  | 2 |  |  |  |  |  |  |  |  | 2 |
| Bay018 |  |  |  |  | 3 |  |  |  |  |  |  |  |  | 3 |
| Bay019 |  |  |  |  |  | 2 |  |  |  |  |  |  |  | 2 |
| Bay020 |  | 7 |  |  |  |  |  |  |  |  |  |  | 5 | 12 |
| Bay021 |  |  |  |  |  | 9 |  |  |  |  |  |  |  | 9 |
| Bay022 |  |  |  |  |  | 2 |  |  |  |  |  |  |  | 2 |
| Bay023 |  |  |  |  |  | 3 |  |  |  |  |  |  |  | 3 |
| Bay024 |  |  |  |  |  | 10 |  |  |  |  |  |  |  | 10 |
| Bay025 |  |  |  |  | 2 |  |  |  |  |  |  |  |  | 2 |
| Bay026 |  |  |  | 2 |  |  |  |  |  |  |  |  |  | 2 |
| Bay027 |  |  |  |  |  |  |  |  | 4 |  |  |  |  | 4 |
| Bay028 |  |  |  |  |  | 2 |  |  |  |  |  |  |  | 2 |
| Bay029 |  |  |  |  |  | 3 |  |  |  |  |  |  |  | 3 |
| Bay030 |  |  |  |  |  | 2 |  |  |  |  |  |  |  | 2 |
| Bay031 |  |  |  |  |  | 5 |  |  |  |  |  |  |  | 5 |
| Bay032 |  |  |  |  |  | 2 |  |  |  |  |  |  |  | 2 |
| Bay033 |  |  |  |  |  |  |  |  |  | 3 |  | 45 |  | 48 |
| Bay034 |  |  |  |  | 31 |  |  |  |  |  |  |  |  | 31 |
| Bay035 |  |  |  |  |  | 3 |  |  |  |  |  |  |  | 3 |
| Bay036 | 6 |  |  |  |  |  |  |  |  |  |  |  |  | 6 |
| Bay037 |  |  |  |  |  |  |  | 41 |  |  |  |  |  | 41 |
| Bay038 |  |  |  |  |  |  |  |  |  | 9 |  |  |  | 9 |
| Ungrouped | 1 |  | 1 | 8 | 19 | 54 |  |  | 6 |  |  |  | 5 | 94 |
| Total | 7 | 7 | 36 | 14 | 77 | 111 | 21 | 46 | 17 | 12 | 11 | 45 | 15 | 419 |

1. See Supplementary Fig. 2
2. Clades are numbered from top to bottom as they appear in the mrBayes output tree available as Supplementary Material.
