# Supplementary material for: Haplotype Affinities Resolve a Major Component of Goat (Capra hircus) MtDNA D-Loop Diversity and Reveal Specific Features of the Sardinian Stock
Source: PLoS One. 2012 Feb 17;7(2):e30785. doi: 10.1371/journal.pone.0030785 (PMC3281868; doi:10.1371/journal.pone.0030785)
Supplement: Table S3 — Representation of sequences of different NJ major clades according to breed (only unambiguous breed assignment) and region. (DOC) [file pone.0030785.s007.doc]

Supplementary Table 3. Representation of sequences of different NJ major clades according to breed (only unambiguous breed assignment) and region

|  |  | NJ Clade | | | | | | | | | | | | |  |
| --- | --- | --- | --- | --- | --- | --- | --- | --- | --- | --- | --- | --- | --- | --- | --- |
| BREED | REGION | Haplogroup C | A1 | A2 | A3 | A4 | A5 | A6 | A7 | A8 | A9 | A10 | A11 | Uncl. | Total |
| Not recorded | GERREI |  |  | 1 |  | 2 | 3 |  | 1 | 2 | 1 | 1 | 2 |  | 13 |
|  | IGLESIENTE |  |  |  | 1 | 1 | 6 | 4 | 4 | 2 |  |  |  |  | 18 |
|  | OGLIASTRA |  |  | 1 |  |  | 2 |  | 1 |  |  |  |  |  | 4 |
|  | SARRABUS | 2 |  | 18 | 5 | 19 | 6 | 3 | 5 | 8 | 1 |  | 15 | 2 | 84 |
|  | SULCIS |  | 1 | 9 |  | 12 | 16 | 4 | 9 | 3 |  | 1 | 7 |  | 62 |
| ALPINA | OGLIASTRA |  |  |  | 1 |  |  |  |  |  |  |  |  | 1 | 2 |
|  | SULCIS |  |  |  |  |  |  |  |  | 1 |  |  |  |  | 1 |
| MALTESE | GERREI |  | 1 |  |  | 3 | 1 |  |  |  |  |  |  | 1 | 6 |
|  | IGLESIENTE |  |  |  |  | 1 | 1 |  | 1 |  |  |  |  |  | 3 |
|  | OGLIASTRA | 1 | 2 | 1 |  | 2 | 19 | 2 | 9 |  |  |  | 5 | 2 | 43 |
|  | SARRABUS |  |  |  |  | 1 | 2 |  | 1 |  |  |  |  |  | 4 |
|  | SULCIS |  |  |  |  |  | 4 |  |  |  |  |  |  |  | 4 |
| SAANEN | GERREI |  |  |  |  |  |  |  | 2 |  |  |  |  |  | 2 |
|  | IGLESIENTE |  |  | 2 |  |  |  |  |  |  |  |  |  |  | 2 |
|  | OGLIASTRA |  |  | 3 |  | 4 | 2 | 2 | 1 |  |  |  | 1 |  | 13 |
|  | SULCIS |  |  |  |  |  | 4 |  | 2 |  | 1 |  | 1 | 1 | 9 |
| SARDA | GERREI |  | 2 | 15 | 8 | 11 | 18 | 6 | 15 | 2 | 2 | 3 | 20 | 3 | 105 |
|  | IGLESIENTE | 3 |  | 6 | 1 | 10 | 12 | 3 | 7 |  | 2 |  | 6 | 1 | 51 |
|  | OGLIASTRA | 1 |  | 48 | 52 | 138 | 63 | 7 | 71 | 32 | 5 | 21 | 27 | 5 | 470 |
|  | SARRABUS | 1 |  | 6 | 2 | 2 | 4 | 1 | 7 | 2 | 1 | 2 | 5 |  | 33 |
|  | SULCIS | 1 | 1 | 33 | 3 | 61 | 56 | 8 | 37 | 28 | 1 | 5 | 19 | 4 | 257 |
| SPAGNOLA | SULCIS |  | 1 |  |  | 1 | 7 |  | 3 |  |  |  | 1 | 1 | 14 |
| Total |  | 9 | 8 | 143 | 73 | 268 | 226 | 40 | 176 | 80 | 14 | 33 | 109 | 21 | 1200 |
