# Supplementary material for: Haplotype Affinities Resolve a Major Component of Goat (Capra hircus) MtDNA D-Loop Diversity and Reveal Specific Features of the Sardinian Stock
Source: PLoS One. 2012 Feb 17;7(2):e30785. doi: 10.1371/journal.pone.0030785 (PMC3281868; doi:10.1371/journal.pone.0030785)
Supplement: File S4 — Summary of GenBank searches for identity/similarity to the most frequent mtDNA haplotypes found in this work. (DOC) [file pone.0030785.s012.doc]

Clade A1 consisted of 7 haplotypes, all of which uncommon (12 sequences). The most common sequence in this clade (NJ085647; n=4) was 98% similar to the reference haplogroup A sequence for Italy (Table 5 in ref. (Naderi et al. 2007)).

Clade A2 consisted of 36 haplotypes, with a clearly modal haplotype (JN085582; 79) identical to isolates SRI, SRH, SRA and M3 (FJ571579, FJ571580, FJ571582, FJ571534) (Vacca et al. 2010).

Clade A3 was a sister clade of A2 and grouped 14 haplotypes. Also in this clade a modal haplotype (JN085583; 40) was found, which was identical to isolate CH519 (EF618119) (Naderi et al. 2007) and to EF618119 sampled in Continental Italy.

Clade A4 consisted of 77 haplotypes, two of which were represented 52 and 46 times, respectively. The first one (JN085612) found no identity, whereas the second one (JN085909) was identical to isolates SRV and SRP (FJ571585, FJ571591) (Vacca et al. 2010). Clade A4 was the mostly represented clade in our set (336 sequences).

Clade A5 grouped the highest number of haplotypes (111) but not of sequences (330). Three haplotypes were represented 20 or more times. Haplotype JN085747 (21) was identical to EF618183 and EF618184, sampled in Italy. Haplotype JN085716

(25) was identical to isolate ChTo2996 (AJ317647) (Luikart et al. 2001) and isolates CH794 CH586 (EF618394.1, EF618186.1) (Naderi et al. 2007), sampled in Spain. Haplotype JN085575 (20) was identical to AJ317735, EF617710, EF617758 and EF618178 sampled in Portugal, Denmark, France and Italy, respectively. Many sequences in this clade were 98% similar to the reference haplogroup A sequence for Iran (EF617945, Table 5 in ref. (Naderi et al. 2007)).

Clade A6 included 21 haplotypes, none of which found identity.

Clade A7 included a modal (JN085584; 40) haplotype, identical to those found in several isolates (HQ455442.1, EU910322.1, EF618182.1 from Italy, FJ571596.1, FJ571595.1, FJ571556.1, FJ571532.1) (Luikart et al. 2001; Wu et al. 2009; Vacca et al. 2010). Many sequences in this clade were 98% similar to the reference haplogroup A sequence for Jordan (EF618200, Table 5 in ref. (Naderi et al. 2007)).

Clade A8 consisted of 17 haplotypes, with a modal (JN085857; 29) haplotype with no identity.

Clade A9 was ill defined and grouped haplotypes from three small subclades. It contained a modal haplotype (JN085741; 7) identical to AJ317659, EF618287, EF617730, AJ317625, EF618151, EF618273 from Malta, Portugal, France, Spain, Italy and Poland.

Clade A10 was downstream to A9 and grouped 11 haplotypes which found no identity.

Clade A11 was also downstream to A9 and was a sister clade of A10. There were two modal haplotypes (11 and 10 sequences, respectively). One sequence in this clade (JN085794) was identical EF618115 sampled in Italy and the remaining ones were highly similar to it.

Luikart G, Gielly L, Excoffier L, Vigne J-D, Bouvet J, Taberlet P (2001) Multiple maternal origins and weak phylogeographic structure in domestic goats. Proc Natl Acad Sci USA 98: 5927-5932

Naderi S, Rezaei H-R, Taberlet P, Zundel S, Rafat S-A, Naghash H-R, El-Barody MAA, Ertugrul O, Pompanon F, for the Econogene Consortium (2007) Large-Scale Mitochondrial DNA Analysis of the Domestic Goat Reveals Six Haplogroups with High Diversity. PLoS ONE 2: e1012

Vacca GM, Daga C, Pazzola M, Carcangiu V, Dettori ML, Cozzi MC (2010) D-loop sequence mitochondrial DNA variability of Sarda goat and other goat breeds and populations reared in the Mediterranean area. J An Breed Genet 127: 352-360

Wu Y-P, Guan W-J, Zhao Q-J, He X-H, Pu Y-B, Huo J-H, Xie J-F, Han J-L, Rao S-Q, Ma Y-H (2009) A fine map for maternal lineage analysis by mitochondrial hypervariable region in 12 Chinese goat breeds. Animal Science J 80: 372-380
