# Supplementary material for: Haplotype Affinities Resolve a Major Component of Goat (Capra hircus) MtDNA D-Loop Diversity and Reveal Specific Features of the Sardinian Stock
Source: PLoS One. 2012 Feb 17;7(2):e30785. doi: 10.1371/journal.pone.0030785 (PMC3281868; doi:10.1371/journal.pone.0030785)
Supplement: File S5 — Details of animal husbandry. (DOC) [file pone.0030785.s013.doc]

**DETAILS OF ANIMAL HUSBANDRY, EXPERIMENTATION AND CARE/WELFARE**

Sardinia has a relatively backward farming system, in terms of structural levels and productivity. Three principal farming systems are utilized, ranging from more traditional extensive system with low structural, management and productive levels, to semi-intensive farming, to eventually the best-developed production chain (Usai *et al*. 2006). In areas sampled in this work (see methods), mostly mountainous and hilly, the prevalent farming system is the extensive method. This situation is particularly evident in Ogliastra sub-region, whereas intensive farming is common in plains as in Oristano area (not sampled). In extensive farming the level of mechanization is very low, grazing is on spontaneously growing plants, and fodder cultivation is a recent introduction.

The supply is mainly based on exploitation of pastures, in woods and meadows. The move from the plains to the mountains, where the late spring and summer are spent, is very common. This allows the rational use of available resources, but results in the difficulty of setting up permanent structures and large farms. Approximately 40% of farms have shelters for animals, while in other cases animals live outdoors all year, in a wild state. This situation is more frequent at with increasing altitude.

Usai MG, Casu S, Molle G, Decandia M, Ligios S, Carta A. (2006) Using cluster analysis to characterize the goat farming system in Sardinia. Livestock Science, 104, 63–76.
